# Supplementary material for: Observation of magneto-electric rectification at non-relativistic intensities
Source: Nat Commun. 2020 Oct 20;11:5296. doi: 10.1038/s41467-020-19125-w (PMC7576171; doi:10.1038/s41467-020-19125-w)
Supplement: Supplementary file 1 — Supplementary Information [file 41467_2020_19125_MOESM1_ESM.pdf]

Supporting Information

Observation of Magneto-Electric Rectification at Non-relativistic Intensities

M. Tuan Trinh[1,2*], Gregory Smail[3], Krishnandu Makhal[1], Da S. Yang[4], Jinsang Kim[4], and Stephen C. Rand[1,3]

[1] *Dept. of Electrical Engineering & Computer Science, University of Michigan, Ann Arbor, MI 48109, USA*
[2] *Department of Physics, University of South Florida, Tampa, FL 33620, USA*
[3] *Division of Applied Physics, University of Michigan, Ann Arbor, MI 48109, USA*
[4] *Department. of Materials Science, University of Michigan, Ann Arbor, MI 48109, USA*
[*] *tm4@usf.edu*

1. Theory of magneto-electric rectification

Both classical and quantum models have successfully described induced magneto-electric rectification in individual diatomic molecules[1]. At moderate intensities (non-relativistic), the nonlinear interaction of the electric and magnetic fields of light gives rise to a static electric dipole moment along the propagation direction. In this dual field interaction, the first photon induces electric polarization at the optical frequency and the second photon exerts magnetic torque on the excited state of the molecule, converting orbital angular momentum to rotational angular momentum that results in magneto-electric rectification.

Classically, the magneto-electric response can be described using a simple Lorentz oscillator model that includes molecular rotations (librations) of a diatomic molecule. A detailed description of the model has been published elsewhere [1,2]. Here, we briefly summarize the classical model before utilizing it to predict the rectification signal observed in this work. The Lorentz force and the corresponding equation of motion for a bound electron subjected to optical fields **E**, **B** are:

$$\mathbf{F}(t) = -e(\mathbf{E} + \mathbf{v} \times \mathbf{B}), \tag{1}$$

$$m\frac{d^2\boldsymbol{\xi}(t)}{dt^2} = \mathbf{F}(t) - m\gamma\frac{d\boldsymbol{\xi}(t)}{dt} - m\omega_0^2(\boldsymbol{\xi}(t) - \mathbf{r}_\mathrm{A}), \quad (2)$$

where $\boldsymbol{\xi}(t)$ is the electron position at time t and $\mathbf{r}_\mathrm{A}$ is the position of the point of equilibrium. Both coordinates are specified with respect to the center-of-mass. ω_0 and γ are the resonant frequency and damping constants of the Hooke's Law oscillator. e and m are the charge and the effective mass of electron, respectively. $\mathbf{F}(t)$ is the total force caused by the electric $\mathbf{E}$ and magnetic $\mathbf{B}$ optical fields.

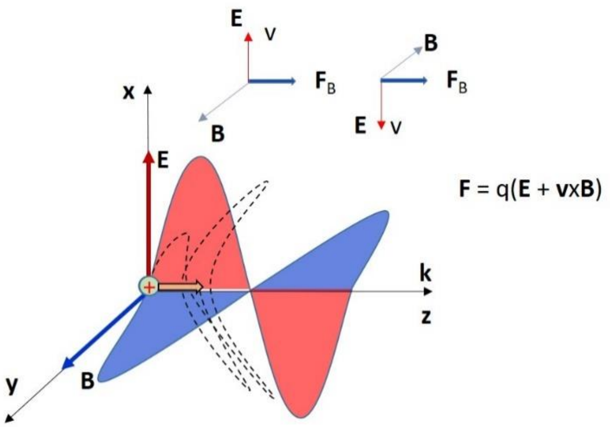

Fig. S1. A schematic of the dual interaction of electric and magnetic field components of light in term of Lorentz force. The magnetic force always points along the propagation direction. The dashed curve presents a trajectory of charge movement in the (x, z) plane over two carrier wave cycles.

Molecular librations are modelled by treating the system as a rigid rotor, so the position of the point of equilibrium becomes time-dependent. Because the radius of rotation is fixed, the equation of motion for the point of equilibrium takes the form of a torsional oscillator,

$$I\frac{d^2\boldsymbol{\theta}(t)}{dt^2} = \mathbf{T}(t) - I\gamma_\mathrm{rot}\frac{d\boldsymbol{\theta}(t)}{dt} - I\omega_\mathrm{m}^2\boldsymbol{\theta}(t), \quad (3)$$

where $\boldsymbol{\theta}(t)$ is the angle between the position vector of the point of equilibrium at time t and its original position. ω_m and γ_rot are the resonant frequency and damping constants of the rigid rotor. I is the moment of inertia of the molecule. $\mathbf{T}(t)$ is the external (optical magnetic) torque acting on

the angular momentum of the rigid rotor. The change in position of the equilibrium point at $\mathbf{r}_\mathrm{A}$ is determined by the angular velocity $\boldsymbol{\Omega}$ through the equation

$$\frac{d\mathbf{r}_\mathrm{A}(t)}{dt} = \boldsymbol{\Omega}(t) \times \mathbf{r}_\mathrm{A}(t). \qquad (4)$$

The only force that acts on the point of equilibrium is equal in magnitude and opposite in sign to the Hooke's Law force in the equation of motion of the electron, by Newton's 3rd Law. The torque exerted on the rigid rotor by this force is

$$\mathbf{T} = \mathbf{r}_\mathrm{A} \times (m\omega_0^2(\boldsymbol{\xi}(t) - \mathbf{r}_A)) = m\omega_0^2 \mathbf{r}_\mathrm{A} \times \boldsymbol{\xi} \qquad (5)$$

In gases or liquids comprised of small molecules, this torque couples electronic excitations to molecular rotations. In solids, where full rotations are not possible, the torque instead causes bound electrons to librate about the equilibrium point in the localized potential within a luminescent center. In this case, librational motion has a natural frequency determined by the azimuthal slope of the local potential, rather than any rotational motion of larger clusters of charges. For this reason the model is extendable from the description of small molecules in liquids to induced charge motion in bulk solids. The equations of motion (2)-(5) were solved using a Matlab differential equation solver. The electron position $\boldsymbol{\xi}(t)$ and the corresponding equilibrium position $\mathbf{r}_\mathrm{A}$ at the (n+1)th time step were calculated from the previous time step. The force and torque were then updated and used to calculate the next time step. The induced electric polarization can then be calculated from $\boldsymbol{\xi}(t)$ using the definition of a dipole,

$$\mathbf{P}(t) = e\boldsymbol{\xi}(t), \qquad (6)$$

and its quasi-static component along the propagation axis can be computed.

Figure S2 presents the evolution of the rectification field resulting from continuous and pulsed laser excitations under various conditions. For pulsed excitation, the rectification field continues

to build up after the pulse due to the momentum of the charge (fig. S2b). The rise and the decay time of the rectification field depend on the pulse duration (Fig. S2c) and the material properties such as rotational/librational frequency and damping parameters (Fig. S2d).

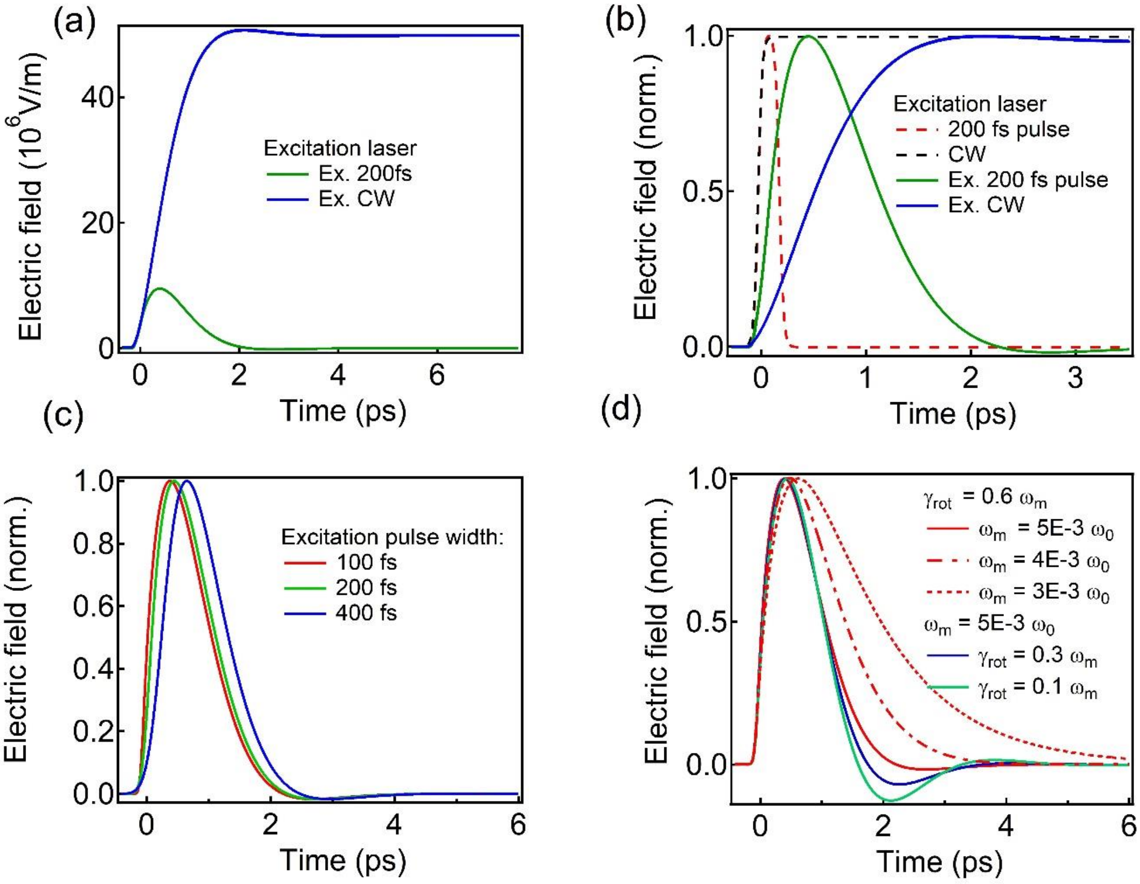

Figure S2. (a) Time dependence of the induced rectification field along the propagation axis for continuous-wave (CW) excitation (blue) and a 200 fs pulse (green). The light intensity is 10^8 W/cm^2. Assumed parameters for a pentacene thin film were ω_0= 4.6×10^{14} Hz, ω = 3.74×10^{14} Hz (laser light frequency, ~ 800 nm), γ = 3.4×10^{13} Hz. The rigid rotor parameters were adjusted to match experimental data, with ω_{m}=5×$10^{-3}\omega_0$, γ_{rot}= 0.6ω_{m}. (b) Pulsed (dashed red) and CW (dashed black) laser profiles together with the corresponding, normalized rectification field amplitudes. (c) Dependence of the rectification field on pulse duration. (d) Theoretical dependence of the rectification field on libration frequency and damping for a 100 fs pulse.

2. Pulse-front tilt characterization

To find the zero of pump-probe delay and to measure the correlation between pump and probe pulses in cross-beam geometry, we placed a GaAs wafer at the sample position and monitored the sum frequency generation (SFG) signal along the bisector of the two beams while translating the delay stage, Fig. S3. The tilt angle was controlled by rotating the grating with respect to the incident laser beam. Since a cylindrical concave mirror was used, the angular direction of the laser beam was little affected by the adjustment of the grating. However, lateral displacement of the beam had to be corrected to pass through two alignment irises using a pair of mirrors.

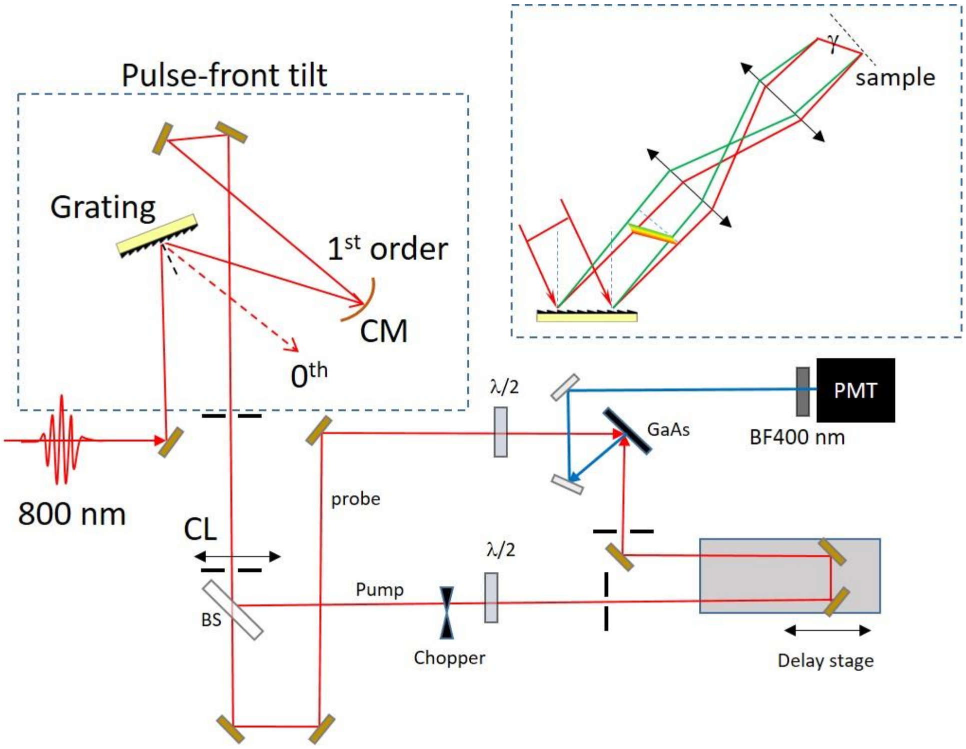

Figure S3. Schematic of the crossed beam pump-probe experiment. The tilted pulse-front was controlled by selecting the grating angle. To find zero delay the sum-frequency generation (SFG) signal was detected with a PMT positioned on the bisector of the incident pump and probe beams in reflection mode (GaAs is opaque at 800 and 400 nm). A 10 nm bandpass filter centered at λ = 400 nm was used to isolate the SFG signal. Top-right box: An illustration of the paths followed by red-shifted and green-shifted components of the pulse after dispersion by the grating resulting in a pulse front tilt angle γ at the sample. Two lenses are needed to correct for the chirp. In the actual setup, a concave mirror (CM) and a cylindrical lens (CL) were used for this purpose.

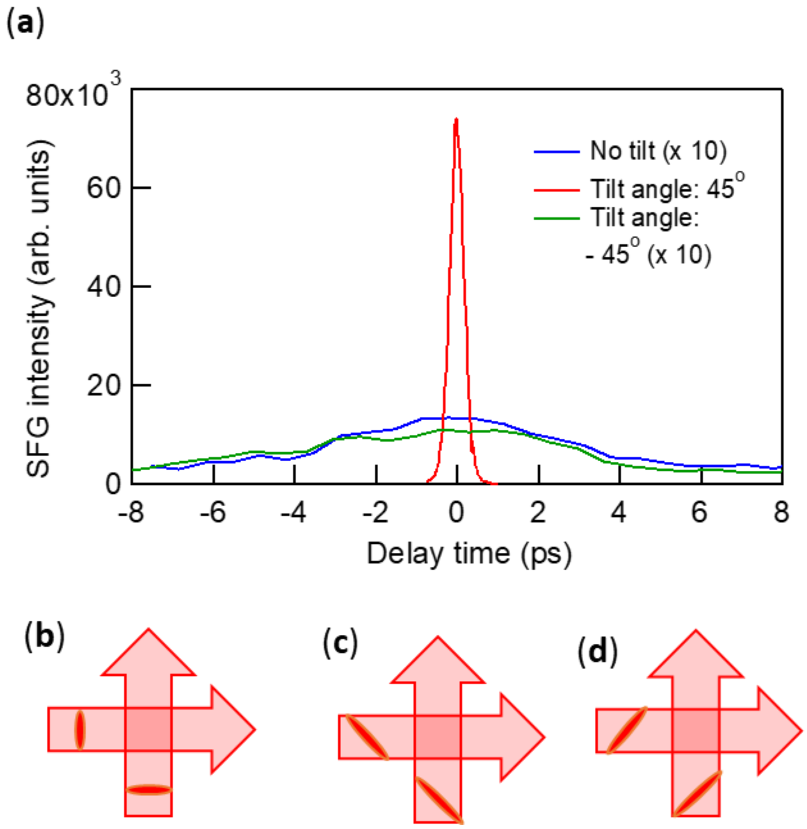

Figure S4. (a) Cross-correlation data for the pump and probe pulses in the crossed-beam experiment. Blue, red, and green curves are for no tilt, tilt pulse front angle at 45, and -45 degrees, corresponding to figures (b), (c), and (d), respectively. (b-d) Illustrations of three possible choices of tilt angle for pump and probe pulses in the crossed-beam geometry. Only (c) provides temporal resolution equal to the pulse duration.

3. Magneto-electric, field-induced second harmonic generation (ME-FISH)

The total second harmonic intensity generated by a probe pulse (propagating in the x direction) includes contributions from the surface, SSHG, and the symmetry breaking, ME-FISH, processes. The latter comes from four-wave mixing process: $\chi^{(3)}\mathbf{E}(\omega)\mathbf{E}(\omega)\mathbf{E}(0)$. These contributions are in phase because both are formed from the incident probe wave. Hence the total intensity is given by:

$$I(2\omega) = \left|\mathbf{E}^{\mathrm{SSHG}}(2\omega)+\mathbf{E}^{\mathrm{ME-FISH}}(2\omega)\right|^2/\eta$$

where η is the electromagnetic impedance of the medium.

$$I(2\omega) \propto \left|\chi_{\mathrm{S}}^{(2)}\mathbf{E}(\omega)\mathbf{E}(\omega) + \chi^{(3)}\mathbf{E}(\omega)\mathbf{E}(\omega)\mathbf{E}(0)\right|^2$$

$$I(2\omega) = a\left|\chi_{\mathrm{S}}^{(2)}\right|^2 I^2(\omega) + b\left(\chi_{\mathrm{S}}^{(2)*}\chi^{(3)} + \chi_{\mathrm{S}}^{(2)}\chi^{(3)*}\right)I^2(\omega)E(0) + c\left|\chi^{(3)}\right|^2 I^2(\omega)E^2(0)$$

where *a, b,* and *c* are constants that depend on geometry. $\chi_{\mathrm{S}}^{(2)}$ and $\chi^{(3)}$ are the surface and the third order susceptibilities, respectively. We can now replace $\chi^{(3)}E(0)$ by $\chi_{\mathrm{eff}}^{(2)}$, the effective magneto-electric susceptibility for second harmonic generation, mediated by the pump-induced ME rectification field.

In our experiment, the pump beam propagates along the z-axis, which is also the rectification field direction. Note that inversion symmetry is only broken by the MER field for input field components polarized along the z-axis. The ME-FISH polarization therefore arises as a DC Kerr-induced signal polarized parallel to the symmetry-breaking rectification field according to

$$P_{\mathrm{z}}^{(3)}(2\omega) = \varepsilon_0\chi_{\mathrm{eff}}^{(2)}\, E_{\mathrm{z}}(\omega)E_{\mathrm{z}}(\omega)$$

where

$$\chi_{\mathrm{eff}}^{(2)} = \chi_{\mathrm{zzzz}}^{(3)}E_{\mathrm{z}}(0)$$

in agreement with Eq. (2) in the main text, and

$$P_{\mathrm{y}}^{(3)}(2\omega) = 0.$$

Hence the total harmonic intensity is

$$I(2\omega) \propto \left|P_{\perp}^{\mathrm{SSHG}}\right|^2 + 2\left|P_{\perp}^{\mathrm{SSHG}} \cdot P_{\mathrm{z}}^{(3)}\right| + \left|P_{\mathrm{z}}^{(3)}\right|^2$$

Note that the first term in this expression is not pump-induced, whereas the second is. The third term can be ignored since it is second order in the (small) rectification field $E(0)$.

The dependence of second harmonic intensity on the probe polarization angle α is different for the pump-independent SSHG and pump-induced ME-FISH signals. Noting that probe

components along y and z are given by $E_{\mathrm{y}}(\omega) = E_0\cos\alpha$ and $E_{\mathrm{z}}(\omega) = E_0\sin\alpha$, respectively, the two contributing nonlinear polarizations are found to vary with α according to

$$P_{\mathrm{z}}^{(3)}(2\omega) = \epsilon_0\left\{\chi_{\mathrm{zyzy}}^{(3)}E_{\mathrm{y}}(\omega)E_{\mathrm{z}}(0)E_{\mathrm{y}}(\omega) + \chi_{\mathrm{zzyy}}^{(3)}E_{\mathrm{z}}(0)E_{\mathrm{y}}(\omega)E_{\mathrm{y}}(\omega)\right\} +$$

$$+\epsilon_0\left\{\chi_{\mathrm{zyyz}}^{(3)}E_{\mathrm{y}}(\omega)E_{\mathrm{y}}(\omega)E_{\mathrm{z}}(0) + \chi_{\mathrm{zzzz}}^{(3)}E_{\mathrm{z}}(0)E_{\mathrm{z}}(\omega)E_{\mathrm{z}}(\omega)\right\} = \epsilon_0\chi_{\mathrm{zzzz}}^{(3)}E_{\mathrm{z}}(0)E_0^2(\omega)$$

and

$$P_{\perp}^{\mathrm{SSHG}}(2\omega) = \epsilon_0\left\{\chi^{(\mathrm{S})}E_{\mathrm{z}}(\omega)E_{\mathrm{z}}(\omega)\cos^2 45°\right\} = \frac{1}{2}\epsilon_0 E_0^2\sin^2\alpha$$

Ultimately, the two leading terms contributing to second harmonic intensity have angular variations with probe polarization that are described by

$$\left|P_{\mathrm{z}}^{\mathrm{SSHG}}(2\omega)\right|^2 \propto I_0^2\sin^4\alpha$$

and

$$\left|P_{\mathrm{z}}^{\mathrm{SSHG}}(2\omega)P_{\mathrm{z}}^{(3)}\right| \propto I_0^2\sin^2\alpha\ .$$

4. Exclusion of electric quadrupole interactions

In addition to the magneto-electric interactions, quadrupolar electric interactions can theoretically give rise to a second order nonlinear response. Consequently, it might be thought that such an interaction could explain rectification in the experiments reported in this work. However, this is not the case. Quadrupole interactions can lead to frequency-doubling but not to rectification, as we show below. Consider an x-polarized pump field $E_{\mathrm{x}} = E_{0\mathrm{x}}e^{i(\omega t - kz)} + c.c.$. A second order polarization that points along the pump propagation axis (z) and results from a quadrupole interaction [3] then has the form

$$P_{\mathrm{z}}^{(\mathrm{q})} = \varepsilon_0\chi_{\mathrm{zxzx}}^{(\mathrm{q})}E_{\mathrm{x}}\frac{\partial}{\partial z}E_{\mathrm{x}}$$

In an isotropic material the tensor susceptibility element $\chi^{(q)}_{zxzx}$ does not vanish. However, upon substitution of the pump field, the nonlinear polarization is found to be

$$P_z^{(q)} = \varepsilon_0 \chi^{(q)}_{zxzx}\{(E_{0x}e^{i(\omega t-kz)} + c.c.)(-ikE_{0x}e^{i(\omega t-kz)} + c.c.)\}$$
$$= \varepsilon_0 \chi^{(q)}_{zxzx}\{-ikE_{0x}^2 e^{2i(\omega t-kz)} + c.c.\}$$

This nonlinear polarization consists exclusively of second harmonic terms. The static field terms vanish. Hence the quadrupole interaction does not support rectification. Moreover harmonic radiation from this interaction yields no SHG signal from the probe alone because the polarization cannot radiate along the propagation axis of the probe, the direction in which the detector is located. Even if the quadrupolar SHG from the pump alone could reach the detector directly, it would not give rise to a pump-induced change in SHG from the probe, but would instead contribute to the total background. Therefore quadrupole interactions cannot account for the pump-induced harmonic probe signal (ΔSHG) in our experiments.

5. Exclusion of charge liberation by electron-hole pair excitation

The pentacene thin film sample in our experiment had an absorption peak at 670 nm, with a tail that extended only as far as 750 nm. The absorption strength at 800 nm, which was the center wavelength of the excitation laser, was therefore negligible. Even so, if electron-hole pairs were excited as the result of absorption at 800 nm and resulted in a charge separation field, the field strength would exhibit a linear dependence on pump power. However, in the present work a quadratic dependence was observed of the pump-induced signal on excitation power.

A weak two-photon absorption could also produce charge carriers, which might cause a SHG signal with a quadratic dependence on pump power. However, the signal rise time would be as fast as the pulse duration in this case, contrary to the observed rise time of ~500 fs. The decay time of

pump-induced SHG would then be either that of the singlet exciton lifetime (24 ns) or the singlet fission time scale (80 fs), See Refs. 28-29 of the main text. Since our signal decay time was ~600 fs, it is quite different from either of these possibilities. Furthermore, we would expect a polarization-independent signal from photo-induced charges, contrary to the experimental results. Our polarization angle dependence was in excellent agreement with the prediction for a magneto-electric rectification field, and distinct from the variation predicted for signals of all-electric origin.

6. Sample preparation and characterization

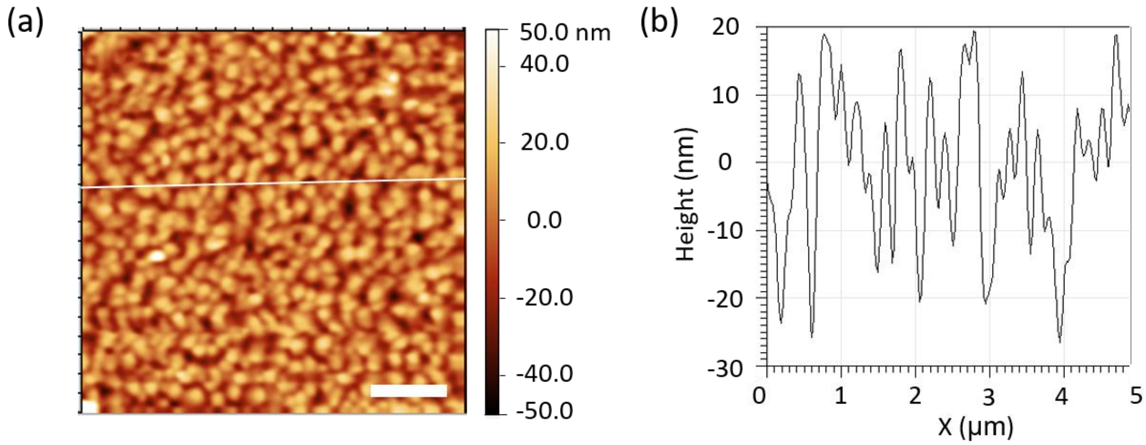

Figure S5. (a) AFM height image of the 400 nm thick pentacene film deposited on glass. The scan size is 5 × 5 μm^2 and the scale bar in the image corresponds to 1 μm. (b) Trace of the variation in height of the pentacene film along the white line in (a). The granularity size is in the range of 100 - 200 nm. The root-mean-square (rms) roughness of the film was 14.82 nm. The AFM image was recorded using Asylum Research MFP-3D AFM in tapping mode with a CT300-25 Aspire probe (spring constant 40 N/m). Surface roughness values were calculated using the Gwyddion software package.

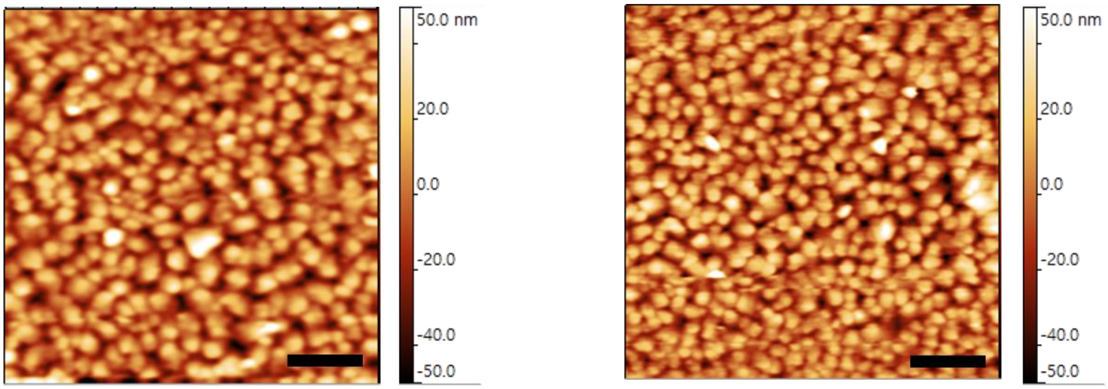

Figure S6. AFM height images of the 400 nm thick pentacene film deposited on glass at two different locations. The black scale bars in the images correspond to 1 μm and the total vertical range of the false color scale is 100 nm.

References

1. Dreyer, E. F. C., Fisher, A. A., Smail, G., Anisimov, P. & Rand, S. C. Optical magnetization, part III: theory of molecular magneto-electric rectification. *Opt. Express* **26**, 17755 (2018).
2. Fisher, W. M. & Rand, S. C. Optically-induced charge separation and terahertz emission in unbiased dielectrics. *J. Appl. Phys.* **109**, 1–8 (2011).
3. P.S. Pershan, Nonlinear Optical Properties of Solids: Energy Considerations. *Physical Review* **130**, 919-929 (1963).
